# Beliefs, perceptions and health-seeking behaviours in relation to cervical cancer: a qualitative study among women in Uganda following completion of an HPV vaccination campaign

**DOI:** 10.3402/gha.v9.29336

**Published:** 2016-02-16

**Authors:** Olivia Topister Hasahya, Vanja Berggren, Douglas Sematimba, Rose Chalo Nabirye, Edward Kumakech

**Affiliations:** 1 Department of Public Health Sciences, Karolinska Institute, Stockholm, Sweden; 2 Gynecological, Breast and Sarcoma Cancer, Department of Oncology, Karolinska University Hospital, Solna, Sweden; 3 Faculty of Health Sciences, Lund University, Lund, Sweden; 4 Makerere University College of Health Sciences, Kampala, Uganda

**Keywords:** beliefs, perceptions, health-seeking behaviours, health belief model, cervical cancer screening

## Abstract

**Background:**

Cervical cancer remains a leading cause of morbidity and mortality in Uganda. Despite earlier information campaigns to introduce human papilloma virus (HPV) vaccination, which also targeted cervical cancer, misinterpretation and misunderstanding of the subject remain high. Women in Uganda present with cervical cancer at an advanced stage due to poor health-seeking behaviours, with an associated high mortality rate. This project explored beliefs, attitudes, perceptions, and health-seeking behaviours in relation to cervical cancer among women in Uganda after an HPV vaccination project had been rolled out.

**Design:**

A qualitative study design was used, with six focus group discussions (FGDs) that included 36 women, aged 25–49 years, with no previous history of cervical cancer symptoms or diagnosis. The women were interviewed in February and March 2013. The transcribed data was analysed using content analysis.

**Results:**

Three themes emerged: feeling unprotected and unsafe, misbelief and wondering about cervical cancer, and fear of the testing procedure. Participating women had heard of cervical cancer but preferred to wait to access cervical cancer screening until symptom debut.

**Conclusions:**

There are still barriers to cervical cancer screening among women in Uganda, where there is a need for culture-specific, sensitive information and interventions to address the issues of improving the cervical cancer screening uptake among these women. Societal context needs to be taken into account when implementing community-based health education.

## Background

Despite being preventable, each year cervical cancer is responsible for about a quarter of a million deaths among women globally, of which about 85% occur in developing countries such as Uganda (www.globocan.iarc.fr). Uganda is a low-income country situated in East Africa with a population of 33.4 million people, 57.7% of which are female (1). The incidence of cervical cancer stands at 52 per 100,000 women of reproductive age, making it one of the highest globally, and more than half of these women die (2, 3). The mortality rate is estimated to be 29.3 per 100,000, and over 80% of diagnosed cases present with an advanced stage of the disease (4). Evidence has shown low uptake and acceptance of cervical cancer screening, with lack of knowledge among the women (5–7).

The screening of women between 35 and 64 years of age for cervical cancer by conventional cytology every 3 to 5 years has shown that invasive cervical cancer can be reduced by at least 80% in European settings (8). Visual inspection with acetic acid (VIA) is the only cervical cancer screening method available at some district hospitals in the rural areas of Uganda (2). This reduces the number of cervical cancer prevention visits and reduces the likelihood of follow-up of the patients (9–11). Several factors have been noted to explain this (12, 13). Although attitudes, beliefs, and perceptions regarding cervical cancer services have been explored in previous studies in Uganda, most were either conducted in regions of Uganda without human papilloma virus (HPV) vaccination programmes or focused on already diagnosed patients and therefore did not properly present the beliefs, perceptions, and health-seeking behaviours among women after HPV vaccine introduction in Uganda, a gap the current study is addressing (14–18).

The HPV vaccination pilot programme in Uganda (19), which was implemented in 2008 through 2009, was targeted at young girls aged 10–14 years and parents, particularly women, aged 25–49 years in the Nakasongola and Ibanda districts. Its services included HPV vaccination, cervical cancer screening, and preventative treatment and sensitisation campaigns. Whereas the programme was implemented by Programme for Appropriate Technology in Health (PATH, a US international health organisation with operations in Uganda), the vaccines were donated by GlaxoSmithKline (GSK, Belgium) and delivery to the girls was funded by the Bill & Melinda Gates foundation. Outcomes of this programme require a qualitative evaluation. The health belief model (HBM) (20) would be of great use in cervical cancer prevention (21, 22). The HBM (Appendix 1, modified by the author) has been used as a psychological model that attempts to explain and predict health-seeking behaviours (20). The model focuses on the attitudes and beliefs of individuals, how they perceive health situations, and the likelihood of changing behaviour in order to prevent a disease outcome (12, 13, 20).

The lack of research in the areas where HPV vaccinations have been implemented demonstrates the importance of conducting qualitative studies in an attempt to explain and predict women's health-related behaviours regarding cervical cancer screening from their own presentation of attitudes and beliefs. This project explored beliefs, attitudes, perceptions, and health-seeking behaviours in relation to cervical cancer among women in Uganda after an HPV vaccination project was rolled out.

## Method

### Study settings

The selection of Uganda as a case for this study was based on its regional participation in the HPV vaccination project. The study was conducted in two rural districts, Nakasongola and Ibanda, found in central and western Uganda, respectively. Ibanda has a population of 250,502, of which half are female (1). In this district, literacy (the ability to read with understanding of basic instructions and to write meaningfully) among adult females is estimated to be 11% and cervical cancer screening attendance is 11%. This attendance percentage reflects the fraction of females aged between 25 and 64 who attend the screening. Nakasongola has a population of 163,600, approximately half of whom are female (1). The female adult literacy rate is estimated to be 66% and cervical cancer screening attendance (the percentage of women 21–64 years of age who received one or more Pap tests) is 7%, according to the 2011 Uganda Demographic and Health Survey (1).

### Study population

Thirty-six Ugandan women aged 25–49 years (Appendix 2: Study Characteristics) were purposively recruited (23) through their daughters who had received HPV vaccination. The inclusion of participants aged as young as 25 years, lower than the minimum age of 30 years recommended by the World Health Organization (2), was due to the high HIV prevalence among younger populations in Uganda, which is known to increase both the risk and progression of cervical cancer. Women aged <25 years and those aged ≥50 years were excluded from the study because the VIA cervical cancer screening method commonly in use in the study setting is associated with unreliable results; therefore these women were not targeted for the VIA-based cervical cancer screening, treatment, and education component of the HPV vaccine programme in Uganda. Moreover, this is a qualitative study whose findings were not meant to be generalisable; selection bias was not expected to apply, as a result of recruiting the mothers of girls who had received HPV vaccination for the study. They were the subgroup of women who came closest to the experience of the HPV vaccination programme and therefore were the most suitable population for understanding women's perceptions and beliefs regarding cervical cancer following the HPV vaccination campaign in Uganda. Women with HIV/AIDS were also excluded from the study because they have their own unique barriers related to disclosure of their HIV status. Moreover, their perceptions, beliefs, and health-seeking behaviours related to diseases in general, let alone cervical cancer, are more likely to be unique from that of their counterparts without HIV/AIDS and therefore deserve a separately tailored study.

In collaboration with the district medical offices, head teachers, and senior mistresses at the schools that participated in the HPV vaccination project, eligible participants with similar characteristics were purposively selected from three rural schools in each of the two districts and formed into focus groups. The eligibility criteria for the study were having an HPV-vaccinated daughter(s), being in the age range of 25–49 years, residing in either the Ibanda or Nakasongola districts, belonging to either the Ankole or Buganda cultural–ethnic groups and being a native speaker of either Runyankore or Luganda, the two most widely spoken local languages in the Ibanda and Nakasongola districts, respectively. Six focus groups comprised six individual participants were formed in order to cover a wide range of experiences (23–25). All participants could speak either Runyankore or Luganda.

### Study design

A qualitative approach was used, with focus group discussions (FGDs) conducted between February and March 2013. An FGD guide was developed and pretested with open-ended questions and used for data collection (Appendix 3: Study questionnaire). The guidelines were cross-checked by the research team. Conducting FGDs was a preferable method as it provided the possibility of dynamic discussions and gave scope for probing during the data collection process (26). This method was used in order to acquire an exploratory understanding of women's beliefs, attitudes, perceptions, and health-seeking behaviours (23–25, 27) on cervical cancer in rural areas 2 years after HPV vaccinations had commenced.

### Ethical considerations

The study was conducted after obtaining ethical approval according to the ethical principles of the World Medical Association. The ethical clearance number SBS-030 was obtained from the Makerere University School of Biomedical Science Institutional Review Board.

### Data collection

The FGDs were held in natural settings that were familiar to the participants, in classrooms where parent meetings were usually held in each school. Each FGD lasted 60 to 90 min. Six FGDs were conducted and moderated by OH, but language clarification in the local dialect was facilitated by the research assistant whenever the need arose. The FGDs were conducted in local languages with which OH was conversant. A pilot discussion was conducted before the onset of data collection. All participants and the research team sat on opposite sides close to each other to minimise the feeling of hierarchy. Discussions were audio-recorded and later transcribed verbatim. Saturation was reached when further FGDs did not add any new findings. In this study, saturation was experienced after three FGDs, but some three more FGDs were carried out in order to confirm saturation. To accomplish confirmability, triangulation and peer review of the collected data were performed and cross-checked with both the participants and the local researcher after every FGD session (23, 25). Saturation was experienced after as few as three FGDs, probably due to the sound approaches used in this study, which involved the use of a theory-based focus group guide; a moderator who was fluent in the local languages for the focus group; ample time of 60–90 min allocated for each focus group; homogeneity among focus group members, which allowed free interactions; heterogeneity between focus groups, which allowed for inclusion of individuals with varied backgrounds and wide experiences, except for the group membership criterion; and assurance of confirmability in the study through procedures such as data triangulation, peer review, and cross-checking with participants and researcher triangulation. Participants were compensated with minimal transport incentives after the interviews.

### Data analysis

The transcripts were read several times and cross-checked by other researchers on the research team. Data were then subjected to qualitative content analysis. They were analysed using latent content analysis, whereby the structural meaning conveyed by the text was extracted. Sub-themes and later three themes emerged after the process. Latent analysis was used in an attempt to acquire an understanding of what the respondents meant (28).

For triangulation, trustworthiness validity, and ensuring dependability, a stepwise replication procedure was used (27). By using a stepwise replication procedure, transcripts were individually read and re-read by three researchers. The researchers then individually built sub-themes, which were compared and revised until an agreement was reached. A similar procedure with periodical discussion by researchers was used for transcripts from all six FGDs. The researchers then revised the outcome until an agreement was reached, interpreted according to Graneheim and Lundman (28).

### Findings

Most participants had heard of cervical cancer and some had been screened. Three themes emerged (Appendix 4) during the analysis: feeling unprotected and unsafe, misbelief and wondering about cervical cancer, and fear of the test procedure.

### Feeling unprotected and unsafe

The first theme was based on the women's feelings of being unprotected and unsafe due to their fear of becoming infected in the health service settings and fear of contracting cervical cancer infection from their husbands. Participants strongly expressed their fears regarding the dangerous, horrible, and incurable nature of cervical cancer. The perception of cervical cancer being spread by men particularly exacerbated their fears.

Fear was also reflected that one could easily contract diseases and get symptoms like bleeding, itching, and pains. Participants also had fears of contracting other diseases in the processes of screening, because they allegedly believed that only one machine was available. They believed that one machine was used from one woman to another without disinfection. They also believed that health workers quite often did not share their findings with clients after diagnosis but instead preferred to keep the findings a secret.

I fear going for a check-up since after getting the diagnosis of cervical cancer, I will then know that I am dying. (FGD4:W2.)

It is an incurable disease brought by men. (FGD3:W3)

Some women used to have normal periods but after going there for screening, they start experiencing abnormal periods, some get itching, the signs. (FGD2:W5)

### Misbelief and wondering about cervical cancer

While most participants believed that cervical cancer screening was important and that cervical cancer was a killer disease, some wondered about the cause and when and why one should be screened before symptom debut. One of the participants who had been screened due to her mother's death from cervical cancer on the contrary advocated cervical cancer screening for all women.

It was emphasised by most participants that a lack of knowledge about cervical cancer explained their poor health-seeking behaviour in relation to cervical cancer screening. The belief and perception that screening could diagnose both cervical cancer and HIV/AIDS made many women decline for fear of knowing their status. Some wondered how their husbands would react if they came home with the news of having been diagnosed with HIV as well.

The participants had contradictory concerns on the purpose and aims of HPV vaccination. Although some believed that vaccinating girls was for their own good, others believed that it was for family planning reasons. For several participants it sounded real that vaccinations were intended to prevent their daughters from producing more than two children in the future.

All women ought to be screened to avoid terrible outcomes. (FGD1:W2)

I have been reluctant to go there. Whoever goes there is checked for AIDS. I do not want to know my status. Then my husband will wonder why I went there at all. (FGD1:W6)

Girls vaccinated during their reproductive age will only get two children. (FGD2: W4)

### Fear of the test procedure

Participants gave several explanations for their failure to attend cervical cancer screening. These were based on past experiences, rumours, fear of male practitioners, economic constraints, and organisational reasons.

In contrast to the few who had undergone cervical cancer screening tests, participants expressed their fears of the testing procedure. The screening procedure was perceived by many as being painful, done with unsterilised equipment, and being embarrassing for one to show private parts to a strange man. In all FGDs, participants spoke strongly in unison about these encounters. They also expressed their concerns about the accessibility and availability of screening centres as essential hindrances to cervical cancer screening. They emphasised that their poor health-seeking behaviour in relation to cervical cancer was also due to having few diagnostic and treatment facilities in the community. Women living in remote rural areas needed to make long and costly journeys to get to screening centres. Concerning availability, participants felt that services were not adequate for all the women who needed to be screened.

The centres were also understaffed and the participants emphasised a wish for more services to be allocated at easily reachable distances, something they said would motivate many women to go for screening.

We are requesting that if they could have the chance of sending health workers down to grassroots, it would be beneficial. (FGD1:W5)

## Discussion

This project aimed to explore the beliefs, attitudes, perceptions, and health-seeking behaviours in relation to cervical cancer among women in Uganda after the HPV vaccination project had been rolled out. The main findings showed that, even though the participants had heard about cervical cancer, they did not have a sufficient understanding of the disease to prompt them to improve their health-seeking behaviour in relation to cervical cancer screening.

Feeling unprotected and unsafe showed an attitude of fear, which was expressed by the participants’ concern about contracting an infection from their husbands and their belief in the possibility of contracting the disease even in the context of cervical cancer screening. The reluctance to go for screening and the fear of getting infections or diseases when being screened for cervical cancer reflected a lack of knowledge about the screening procedure. This was consistent with other findings in earlier studies, where women boycotted screening due to attitudes of fear (29, 30).

Misbelief and wondering about cervical cancer and fears of the test procedure explain respondents’ perceptions of cervical cancer, the barriers to screening, and reasons why they did not attend screening. This is in line with earlier studies, which showed that if women considered barriers to outweigh the benefits of screening, then they had difficulties in complying with screening attendance (29, 31, 32). This raises concern, as emphasised in earlier studies about the importance of knowledge about cervical cancer and its interrelation with higher cervical cancer screening attendance (29, 33–35).

The results of this study show respondents’ emphasis on similar barriers in the form of availability difficulties and lack of knowledge. It is therefore important that health promotion efforts focus on improving women's knowledge of risk factors. That can best be done by providing the women with information about the benefits of early screening, early detection, and its association with lower incidence and mortality rates from cervical cancer (5, 31, 34–36). In order to ensure strong and effective cervical cancer preventive programmes, strategies that focus on cost-effective services and availability and use of the services by the women who need them most are vital. According to a study conducted in low- and middle-resource countries, factors affecting women's rates of cervical cancer prevention uptake included socio-cultural norms, clinical requirements, and the type of services delivered. It also showed that women from rural areas had a limited understanding of female reproductive organs and associated diseases (37). This is in line with the knowledge gap hypothesis, which is a prediction that group information uptake and increased flow of information into a social system (e.g. from a media campaign) are more likely to benefit groups of higher socio-economic status than those of lower socio-economic status (38). The above-mentioned factors are also important in guiding the targeting of strategies – for example, production of educational material and identification of channels through which the audience can be reached (20). Health education materials should be appropriate for and ideally matched to the educational levels of particular targeted audiences (13, 20).

In several studies (36, 39) the HBM has been used to establish the relationship between health beliefs and health-seeking behaviours. The studies showed that women who did not perceive themselves as being susceptible or at risk of contracting cervical cancer had poor cervical smear test attendance (20, 31, 36). In line with other studies on the subject, this study explored beliefs, attitudes, perceptions, and health-seeking behaviours reflecting a lack of knowledge about cervical cancer and cervical cancer screening and the limits on respondents’ perception of their susceptibility to the diseases (20, 40).

The perceived severity of cervical cancer alone without the perceived benefits of early screening, such as in this study, does not motivate women to seek out screening services. The attitude of reluctance or hesitance to go for screening can be explained as a lack of acknowledgment of the fact that they are susceptible to cervical cancer and that they would benefit from screening, as shown in earlier studies (41, 42). In terms of perceived barriers to seeking cervical cancer screening, participants in all FGDs as in earlier studies (5, 40, 43) emphasised lack of knowledge, economic constraints, inaccessibility of health services, cultural beliefs, and the belief that cervical cancer is an incurable disease as hindrances, which calls for further qualitative studies (12, 29, 37, 44).

Applied research and health education should be adapted to the Ugandan rural setting; researchers ought to take into account the methods, strategies, and application of theories that are specifically suitable.

## Conclusion and recommendations

This was a pilot study following an HPV vaccination project, and findings in this study correlated with those of similar studies in the same context with poor cervical screening attendance. It is asserted that individual change will follow successful organisational and environmental changes, provided that these changes are sustained over time. Even after information was disseminated, the women in Ibanda and Nakasongola districts still maintained misguided beliefs, attitudes, and poor health-seeking behaviours in relation to cervical cancer.

Health promotion activities need to be tailored towards improving knowledge levels about the risk factors of cervical cancer and the importance of screening, as well as the impact on mortality due to cervical cancer. Action should be taken to understand their beliefs and perceptions to provide for effective delivery of public health programmes.

This could be promoted by the use of multisectional approaches, addressing culture-specific issues, and provision of sensitive and competent services.

To this end, suitable ways of encouraging uptake, ensuring flexibility of screening appointments, provision of appropriate educational materials, and enhancing the interpersonal linkage between health personnel and clients ought to be considered for the purpose of narrowing the knowledge gap.

### Strengths and weaknesses of the study

Data collected or used in this study were collected in natural settings and participants were free to speak their mind based on experiences and reality. Other strengths of the study were that the main author works with a cervical cancer treatment unit, has worked in Uganda's rural settings, originally comes from Uganda, and is conversant with the languages spoken and the socio-cultural environment.

Although the qualitative focus group design of this study was vital for the research questions, a limitation of this study is that the findings may not be transferrable to other settings due to the cultural and ethnic diversity in Uganda.

## **Appendix 2**. Demographic data

**Table:**

Demographic characteristics		
Age	5	14%
25–29	9	25%
30–34	10	27%
35–39	6	17%
40–44	6	17%
45–49		
Marital status		
Single	6	17%
Married	30	83%
Education level		
Educated, working at professional level	5	14%
Literate	16	44%
Illiterate	15	42%
Children		
0–2	15	42%
3–5 +	21	58%
Ever been screened for cervical cancer?		
No	29	81%
Yes	7	19%

## **Appendix 3**. Focus group discussion questionnaire guide

**Table:**

Summary guide for focus group discussion	
	Questionnaires
**Theme 1**	
**1**	What do people in the community say or think about cervical cancer?
	Review: Reasons, myths, beliefs, attitudes to someone with the disease in the community
**2**	What are your own views about cervical cancer?
	Review: Do you know anybody who has had cervical cancer or who suffers from cervical cancer?
**3**	What do you know about the test done to diagnose cervical cancer?
**Theme 2**	
**1**	What do people say about health services in relation to cervical cancer?
	Review: Do women in the community go for screening?
	What about you/yourselves? Why do or did you go there? Why not?
**2**	What do you think facilitates the health system in relation to cervical cancer?
**3**	What do you think would prevent women from seeking out cervical cancer screening, in case of suspected cervical cancer?
	Review: What about yourself?
**4**	Do you have any personal experience to share?
**Theme 3**	
**1**	What do people think about the vaccination of girls that began taking place almost 2 years ago?
	Review: Concerns about vaccinations, beliefs about vaccinations; what do you yourselves believe and think about the vaccinations?
	Thank you for your contribution; it is very important.

## **Appendix 4**. Chart showing examples of meaning units, condensed meaning units, sub-themes, and themes from content analysis of part of the FGD data

**Table:**

Meaning units (MUs)	Condensed MUs	Sub-themes	Themes
Some women used to have normal periods, but after cervical cancer screening they started experiencing abnormal periods. Some got itching and other signs too. An incurable disease brought by men.	They experienced abnormal periods and acquired symptoms in relation to the screening procedure. Cervical cancer is spread by men.	Fear of contracting cervical cancer.	Feeling unprotected and unsafe.
Those who go for screening are the ones who have had abdominal pain but for me, I have not had pains, so there is no need to go for screening.	Those who have abdominal pain go for screening. There is no need for screening before.	Screening is only done after symptom debut.	Misbelief and wondering about cervical cancer.
We request that health workers be sent to the grassroots level. I am afraid to go there because screening is carried out by a male healthcare practitioner.	Send health workers to the grassroots level. Fear of male healthcare personnel.	Requests for suitable staffing at the grassroots level.	Fear of the test procedure.
